# A cluster randomized trial protocol to evaluate the effectiveness of an integrated package of improved take-home foods complemented with social and behaviour change communication strategies to improve nutritional status in children aged 6–36 months in six states of India: NECCTAR trial

**DOI:** 10.3389/fnut.2026.1712806

**Published:** 2026-03-12

**Authors:** Mrunali Zode, Suparna Ghosh Jerath, Manoja Kumar Das, Arun Kokane, Balamurugan Ramadass, Radhika Madhari, Rebecca Kuriyan, Zahiruddin Quazi Syed, Abhay Gaidhane, Sumathi Swaminathan, Sumithra Selvam, Teena Dasi, Tinku Thomas, Abdul Jaleel, Shital Telrandhe, Raghavendra Rao Chowdavarapu, Kritika Singhal, Vani Kandpal, Susmita Chatterjee, Sudipto Roy, Tanica Lyngdoh, Bharati Kulkarni

**Affiliations:** 1 Indian Council of Medical Research, New Delhi, India; 2 The George Institute for Global Health, New Delhi, India; 3 The INCLEN Trust International, New Delhi, India; 4 All India Institute of Medical Sciences, Bhopal, Madhya Pradesh, India; 5 All India Institute of Medical Sciences, Bhubaneswar, Odisha, India; 6 ICMR—National Institute of Nutrition, Hyderabad, Telangana, India; 7 St. John's National Academy of Health Sciences, Bangalore, Karnataka, India; 8 Datta Meghe Institute of Higher Education and Research, Wardha, Maharashtra, India; 9 Academy of Scientific and Innovative Research, Ghaziabad, Uttar Pradesh, India

**Keywords:** complementary feeding, India, infants and young children feeding, IYCF, nutrition, social behavioural change communication, take home ration, undernutrition

## Abstract

**Background:**

Nutrition during early childhood is critical for growth, development, and long-term well-being, with age-appropriate complementary feeding playing a pivotal role in meeting a child’s nutritional needs. Despite concerted efforts through national programs in India, gaps persist in complementary feeding practices, contributing to the enduring burden of undernutrition. This study aims to address these gaps by developing an intervention package comprising state- and district-specific improved take-home rations (THRs) provided under the Integrated Child Development Services (ICDS) alongside socio-culturally tailored social and behaviour change communication (SBCC) strategies to improve nutrition among children aged 6–36 months.

**Methods:**

The study will be conducted in one selected district from each of six Indian states—Karnataka, Madhya Pradesh, Maharashtra, Meghalaya, Odisha, and Rajasthan. The study comprises three sequential phases. In the first phase, formative research will explore current dietary practices of children aged 6–36 months, including THR uptake, as well as perceptions and challenges related to existing THR provision under ICDS and SBCC initiatives from both supply- and demand-side stakeholders. In the second phase, insights from formative research will inform co-development of an intervention package comprising optimized THR formulations and a multi-level SBCC strategy through an iterative and participatory process, which will be piloted to assess feasibility and acceptability. In the final phase, a two-arm cluster randomized controlled trial (cRCT) will evaluate the effectiveness of the intervention in improving nutritional status and complementary feeding practices compared to existing THR and SBCC strategies. cRCT will comprise: (1) a longitudinal cohort of children aged 6–18 months receiving the intervention for 18 months, with follow-up at baseline, 6, 12, 18, and 24 months to assess outcomes at individual level; and (2) repeated cross-sectional surveys of all children aged 6–36 months residing in study clusters at baseline and 6-month intervals up to 24 months to capture population-level changes.

**Discussion:**

The study will demonstrate the effectiveness of improved THR products combined with socio-culturally relevant SBCC strategies in improving feeding practices and nutritional status of children aged 6–36 months, while also assessing cost-effectiveness. By prioritizing local acceptability, sustainability, and use of locally available nutrient-rich foods aligned with regional dietary preferences, the intervention aims to bridge the gap between policy intentions and community practices. Findings will inform future THR reform and complementary feeding initiatives in India.

**Clinical trial registration:**

https://ctri.nic.in/Clinicaltrials/searchbyctri.php, identifier CTRI/2024/10/075472.

## 1 Background

Nutrition is central to achieving the Sustainable Development Goals (SDGs), with at least 12 of the 17 SDGs incorporating nutrition-related indicators (1). Despite economic growth and numerous policy initiatives, India continues to bear nearly one-third of the global burden of undernutrition (2). Most recent prevalence estimates for stunting (35.5%), wasting (19.3%), and underweight (32.1%) in children under five in India remain high, exceeding World Health Organization (WHO) thresholds for public health concern (>30% for stunting, >15% for wasting) (3).

The first 1,000 days of life represent a critical window for physical and cognitive development, laying the foundation for lifelong health (4). Although the benefits of timely and appropriate complementary feeding during this period are well documented, significant gaps remain between recommended and actual practices, particularly in low- and middle-income countries (LMICs) (5–11). In India, the Integrated Child Development Services (ICDS) scheme through its Supplementary Nutrition Programme (SNP) provides take-home rations (THRs) for children aged 6–36 months and pregnant and lactating women, along with hot cooked meals for preschool children aged 3–6 years, to improve nutritional status (12). However, program impact is often constrained by persistent challenges such as suboptimal food quality, poor complementary feeding practices, inappropriate timing, and inadequate portion sizes (10, 13–16). To address these gaps, there is growing recognition of strengthening social and behaviour change communication (SBCC) components as a critical and complementary strategy in nutrition programs. Evidence shows that integrating SBCC into nutrition programs can enhance caregiver knowledge, promote appropriate feeding behaviours, and improve dietary and nutritional outcomes (17–23).

Recognizing the gaps, NITI Aayog, the Government of India’s apex public policy think tank has advocated for strengthening the delivery of nutritious THRs, improving production, supply chain mechanisms, and SBCC to enhance the effectiveness of ICDS (24). Recent initiatives, such as Saksham Anganwadi and Mission Poshan 2.0, aim to align supplementary nutrition with updated requirements (25). Nevertheless, persistent issues remain, including poor nutrient composition of THRs, intra-household sharing of rations, and limited cultural acceptability (9, 10, 26). The Government of India, in January 2023, revised Schedule II of the National Food Security Act (2013), introducing updated nutrition norms that go beyond energy and protein, incorporating quality of protein, healthy fats, carbohydrates, and seven essential micronutrients (27).

Addressing undernutrition requires interventions that move beyond improving food composition to also consider socio-cultural and behavioural factors influencing feeding practices. Household dynamics, caregiving practices, and community beliefs significantly shape dietary behaviours. Therefore, context-specific, culturally appropriate SBCC strategies that can enable and engage communities are essential to foster uptake and ensure sustained improvements in child nutrition (28–31).

The present study aims to improve the nutritional status of children aged 6–36 months through an integrated approach that enhances the nutritional quality of THRs and promotes complementary feeding practices using tailored SBCC strategies. The study proposes to develop and evaluate the effectiveness of the improved, state- and district-specific THRs in combination with socio-culturally appropriate SBCC strategies to enhance the nutritional status of children aged 6–36 months across selected sites in six Indian states.

Study objectives are defined as:

Primary objective:

1. To evaluate the effectiveness of an integrated intervention package, comprising improved THR and SBCC strategy, targeted at children aged 6–36 months (and their caregiver/households) on weight-for-age z score (WAZ) after 18 months of implementation, compared to the existing THR and SBCC strategy.

Secondary objectives:

2. To evaluate the effect of the intervention package targeted at children aged 6–36 months (and their caretakers/households) compared to the existing THR and SBCC strategy after 18 months of implementation on length/height-for-age z score (LAZ/HAZ), weight-for-height/length z score (WHZ/WLZ), the prevalence of underweight (WAZ < -2SD), wasting (WHZ/WLZ < -2 SD), stunting (LAZ/HAZ < -2 SD), infant and young child feeding practices (IYCF) (such as minimum meal frequency, minimum dietary diversity, and minimum acceptable diet), child morbidity indicators, and caregiver knowledge, skills, practices, and behaviour towards infant and young child feeding practices.
3. To document the incremental cost-effectiveness of the integrated intervention package compared to the existing THR and SBCC strategy in the study districts.

## 2 Methods and materials

This multicenter study will be conducted in three sequential phases over a period of 48 months. The first phase will involve formative research to explore and understand the perceptions, practices, and attitudes of different stakeholders, as well as the challenges related to the existing THR and SBCC efforts for addressing the nutritional status of children aged 6–36 months, using an exploratory cross-sectional design with embedded mixed methods. In the second phase, an improved and context-specific THR and SBCC intervention package will be developed and piloted using a participatory research approach with mixed methods. The final phase will involve a two-arm cluster randomized controlled trial (cRCT) to evaluate the effectiveness of the developed intervention package.

### 2.1 Study setting

**Figure 1:**
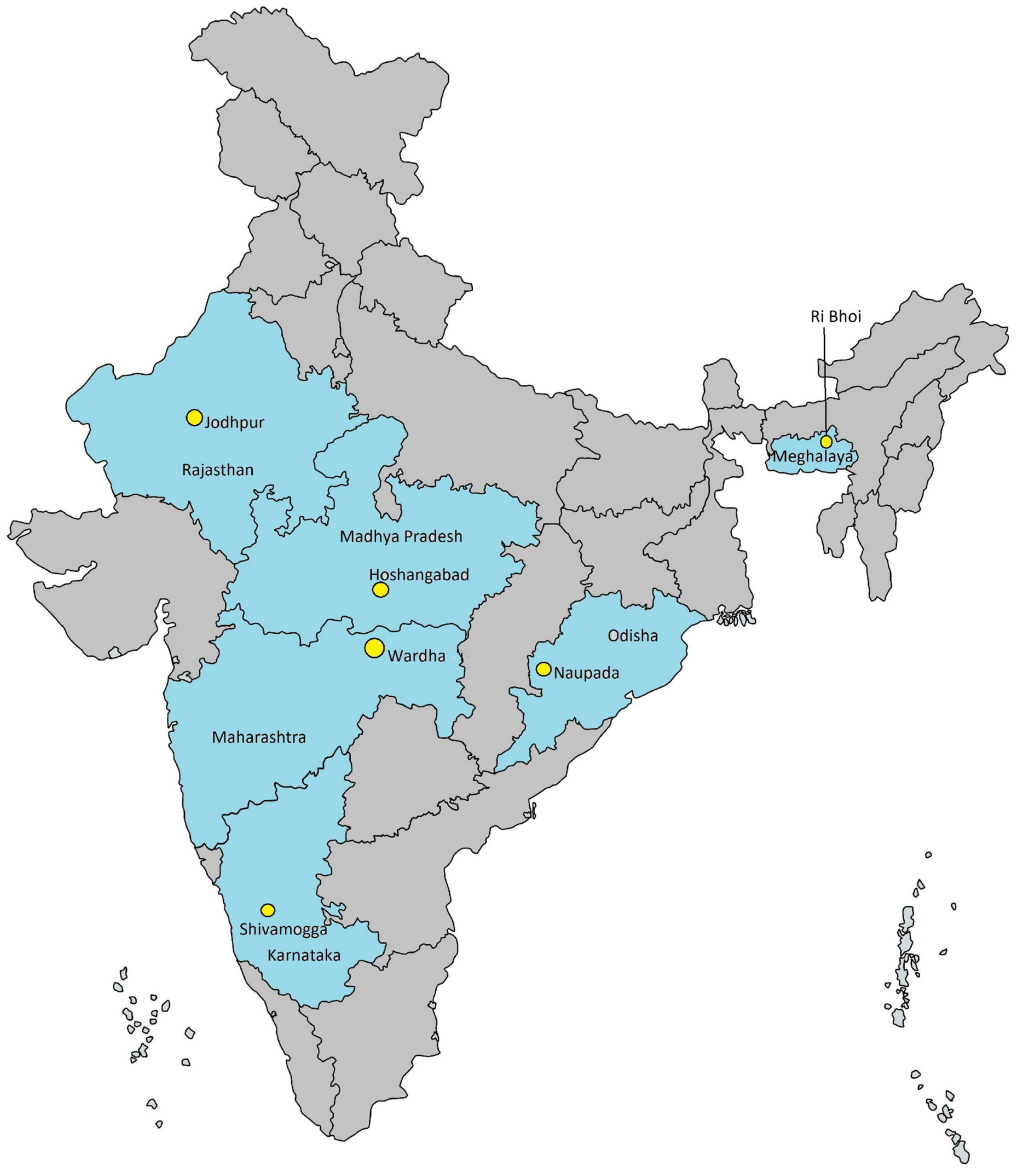
Study sites—selected states and the districts.

All phases of the study will be conducted in one selected district from each of six Indian states, ensuring representation across diverse geographic regions, cultures, and dietary diversity. Study activities at each site will be implemented by a consortium of institutions. Study sites, including selected states and districts, are illustrated in Table 1 and Figure 1.

**Table 1:** The study districts selected for the study.

Region	State	District	Implementing institution
East	Odisha	Nuapada	All India Institute of Medical Sciences, Bhubaneshwar, Odisha, India and The INCLEN Trust International, New Delhi, India
West	Maharashtra	Wardha	JN Medical College, Datta Meghe Institute of Higher Education and Research, Wardha, Maharashtra, India
North	Rajasthan	Jodhpur	ICMR—National Institute of Nutrition, Hyderabad, Telangana, India
South	Karnataka	Shivamoga	St John’s Research Institute, Bengaluru, Karnataka, India
Central	Madhya Pradesh	Hoshangabad	All India Institute of Medical Sciences, Bhopal, Madhya Pradesh, India
Northeast	Meghalaya	Ri Bhoi	The George Institute for Global Health, New Delhi, India

### 2.2 Phase I: formative research

Exploratory research will be undertaken over a period of six to seven months to assess the current status of complementary feeding practices and THR implementation and uptake, and to generate context-specific, evidence-based insights. The research will be driven by four key objectives:(1) to assess the current provisions, quality, implementation, and uptake of THR under ICDS program in selected states; (2) to explore the perceptions of key stakeholder including policymakers, program implementers, frontline workers, vendors, and civil society members regarding the challenges and gaps in the THR products and program implementation; (3) to assess IYCF feeding and dietary practices, including locally prevalent, traditional, and culturally embedded complementary feeding practices, and positive feeding behaviours among children aged 6 to 36 months, and to document perspectives of caregivers and key community stakeholders; and (4) to evaluate the nutritional and chemical profile of the existing THR products. These objectives will be achieved through various activities outlined in Table 2.

**Table 2:** Components of formative research.

Activity	Tools/methods	Key outputs
Review of existing literature	Desk review	THR policies, guidelines, composition, supply chain, SBCC strategies, best global practices, and relevant SBCC theories/models
Mapping of the THR supply chain and key stakeholders engaged in the selected district	Net mapping tool (32); Key informant interviews (KIIs) with the stakeholders identified through stakeholder mapping and snowball sampling.	Current THR provision (ingredients, nutrition profile), end-to-end supply chain from procurement to distribution, logistics and costs, etc.
Observations related to program implementation	Observation checklists; In-depth interviews (IDIs) and KIIs.	Existing training standards, adequacy of personnel, infrastructure, and SBCC strategies.
Qualitative inquiries with supply-side stakeholders	IDIs, focused group discussion (FGDs) (until data saturation).	Perceptions of supply-side actors (program implementers, frontline workers like anganwadi workers (AWWs), anganwadi helpers (AWHs), vendors, civil society members, SHG, etc.) on challenges and gaps in current THR composition and SBCC delivery.
Qualitative inquiries with demand-side stakeholders	IDIs, FGDs, and food free-listing guides.	Caregiver and community perceptions regarding the dietary practices, complementary feeding, children’s food preferences, local and indigenous foods, exploration of locally prevalent positive traditional feeding practices practiced by positive deviant households, acceptability and uptake of current THR and SBCC activities, and their expectations for improvement.
Household survey along with KABP	KABP questionnaire (estimated sample size, *N* = 220 children aged 6–36 months); 24-h dietary recall tool (2 nonconsecutive days).	Individual level—caregivers’ KABP regarding complementary feeding, diet diversity, frequency and quantity of feeding, THR use, nutrition intake of children. Household level—socio-demographic and environmental factors likely to affect complementary feeding and nutritional status of the children.
Market survey	Market survey tool (33)	Price of locally available foods at the community level.
Laboratory analysis of existing THR	Batch-wise macronutrient and micronutrient analysis; FSSAI-certified labs; samples to be sent to a second FSSAI-certified laboratory for quality assurance.	The nutritional, chemical, and microbiological profile following Schedule II of the National Food Security Act, 2013, revised in January 2023 (27).

IDI, In-Depth Interview; KII, Key Informant Interview; FGD, Focused Group Discussion; KABP, Knowledge, Attitudes, Beliefs, and Practices; FSSAI, Food Safety and Standards Authority of India; AWW, Anganwadi Worker; AWH, Anganwadi Helper. Net-Map is a group interview-based tool that helps in understanding the interplay of complex formal and informal networks, stakeholder influence, and power dynamics through visualization exercises. In this study, an adapted Net-Map tool will be used to map the Take-Home Ration (THR) supply chain under the ICDS Supplementary Nutrition Program, identify key actors at policy and implementation levels, and understand their roles, interconnections, and influence across the supply chain.

During this phase, the study will document traditional complementary foods, recipes, preparation methods, feeding norms, and caregiving and feeding practices, and identify positive behaviours through in-depth discussions with caregivers, particularly mothers of well-nourished children from the local community. These insights will inform the contextually appropriate practices that can be highlighted and promoted as part of the social and behaviour change communication strategy of large-scale supplementary feeding programmes. The evidence generated during the formative phase will be instrumental in informing the development of optimized THR compositions and the design of the SBCC strategy tailored to specific state and district contexts, ensuring cultural appropriateness, local relevance, and cost-effectiveness.

### 2.3 Phase II: development and piloting of the improved integrated intervention package

**Figure 2:**
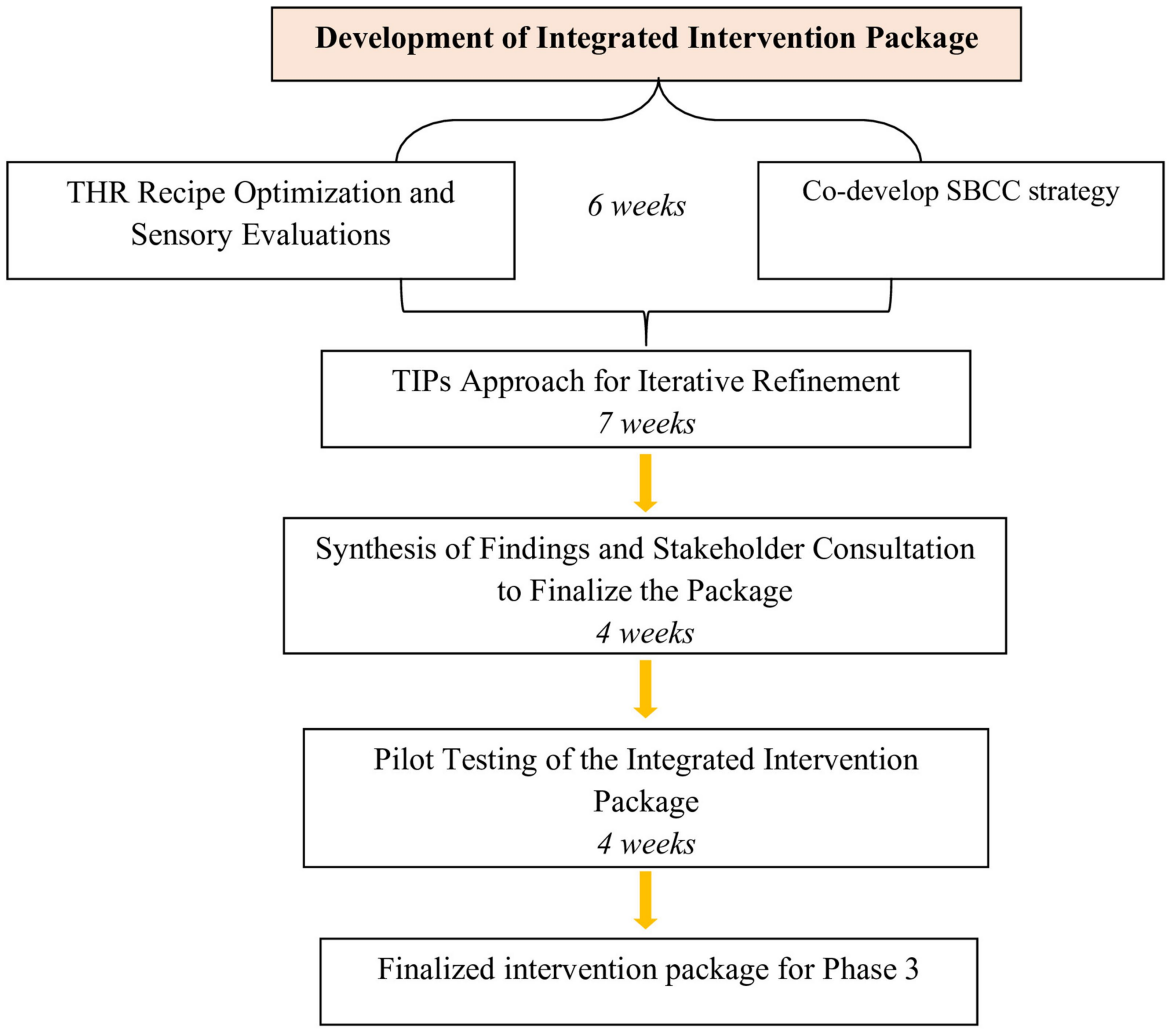
Flow of activities in phase 2.

Phase 2 will span six months and will involve a sequence of activities to develop, refine, and test the intervention package. The flow of activities in phase 2 is illustrated in Figure 2.

#### 2.3.1 THR recipe optimization and sensory evaluations

This step will involve finalizing the THR formulations (2–3 variants) based on nutritional modelling and stakeholder consultations. A systematic process will be undertaken to select ingredients and formulate THR blends that meet energy and nutrient standards (see Supplementary Table 1). Field-level testing will be carried out through cooking demonstrations to assess the palatability, texture, and ease of preparation of the proposed enhanced THR recipes, along with sensory evaluations with caregivers using Likert-type or hedonic scales. Standardized, structured sensory evaluation questionnaires will be administered by trained field investigators immediately following the cooking demonstrations and tasting sessions. These questionnaires will capture caregiver ratings of appearance, aroma, texture, taste, ease of preparation, and overall acceptability for each recipe. Data will be collected at the individual caregiver level using paper-based or electronic forms and entered into a centralized database for analysis. Caregiver feedback on ingredient availability, cooking feasibility, and feeding challenges will be documented. Simultaneously, assessments of existing household practices, including current dietary practices, food access, and cultural beliefs influencing feeding, will also be conducted using structured interviews and food preference ranking exercises. In addition, the nutritional status of the selected children will be assessed.

These activities will yield a refined list of nutritionally enhanced, culturally acceptable THR products and recipes tailored to local food habits and seasonal availability, along with household-specific dietary contexts established for subsequent Trials of Improved Practices (TIPs) engagement.

#### 2.3.2 Co-development of evidence-based, contextual, multi-level SBCC strategy

**Figure 3:**
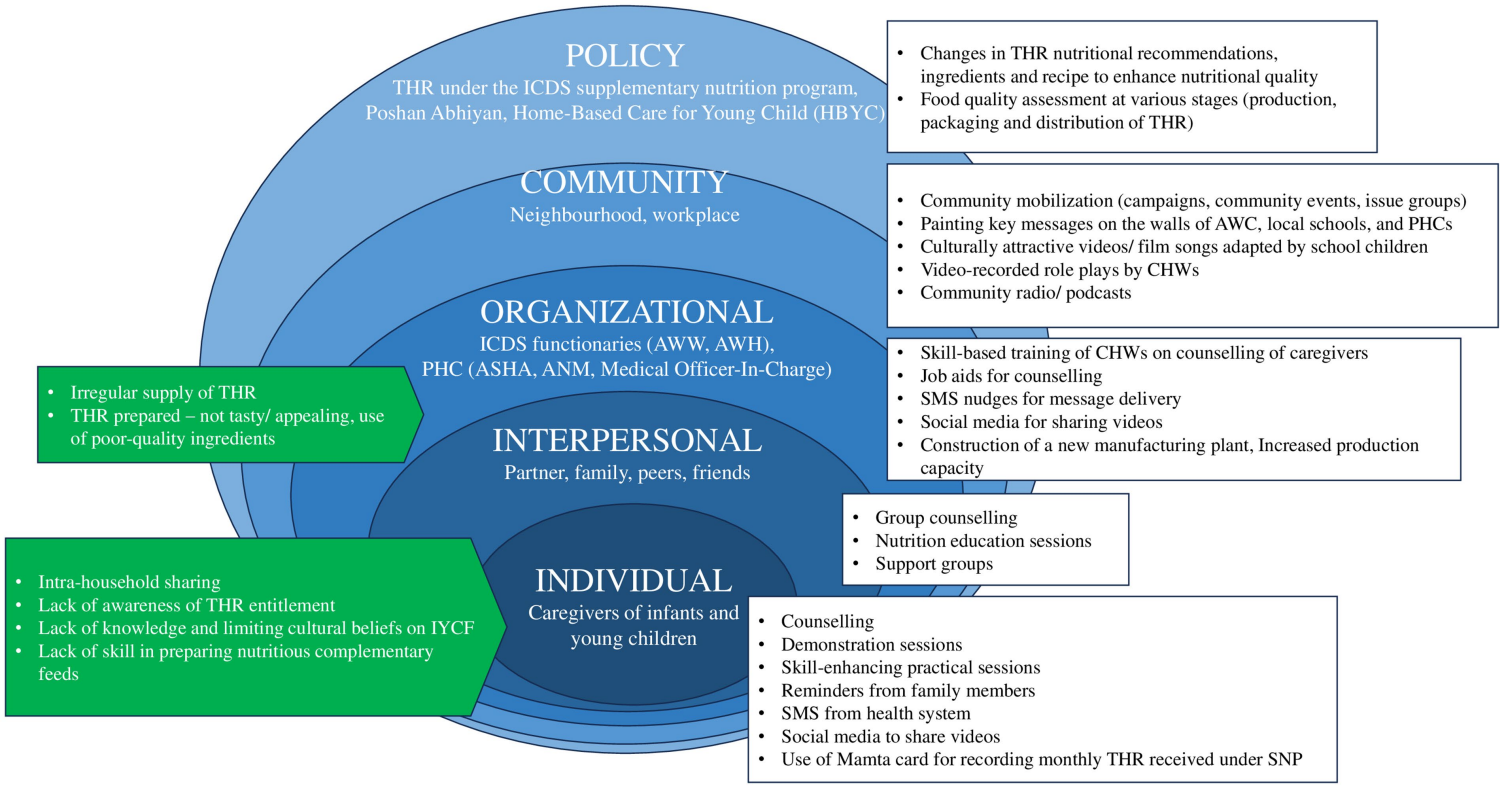
SBCC framework to promote IYCF practices and utilization of take-home rations. Factors shown on the left depict potential reasons for the low consumption of take-home rations (THR) identified in the literature (9, 10, 13, 15, 26, 39, 40) while those on the right represent approaches and strategies reported in studies to promote IYCF practices and enhance supplementary feeding utilization (21, 24, 30, 31, 41–43). This SBCC framework is adapted with permission from UNICEF’s Socio-Ecological Model (34–36).

The social and behaviour change communication strategy, as part of the intervention package, will be developed utilizing the Socio-Ecological Model (34–36). In addition, behavioural theories such as Social Cognitive Theory and Nudge Theory will guide the design of SBCC activities, ensuring that the strategy is both locally acceptable and context-specific by addressing barriers identified during the formative phase (37, 38). Since behaviours are shaped by complex influences at multiple levels—individual, interpersonal, organizational, community, and policy- it is critical that the SBCC strategy addresses determinants across these domains. Previous studies have identified potential reasons for the limited consumption of take-home rations across these levels (10, 13, 15, 26, 39, 40). They have also explored various approaches to promote IYCF practices and improve the uptake of supplementary feeding (21, 24, 30, 31, 41–43). Building on this evidence, the improved strategy will give primary emphasis to behaviour change at the individual and interpersonal levels, focusing especially on caregivers while also engaging relevant stakeholders across higher levels of influence. The SBCC framework to promote IYCF practices and utilization of THR is illustrated in Figure 3.

The enhanced SBCC components will aim to strengthen maternal knowledge and feeding behaviours, thereby improving IYCF practices and use of improved THRs. In addition, they will aim to elevate the capacity and engagement of frontline workers and other key stakeholders.

#### 2.3.3 Trials of improved practices (TIPs) approach for iterative refinement

Five 1-week cycles of TIPs will be conducted with households over the seven weeks to study the feasibility, adherence, acceptability, and willingness to adopt the proposed recipes and SBCC messages (44). Each cycle will provide insights to iteratively adapt the intervention, guided by caregiver feedback and local constraints. Field staff will engage with selected households (approximately 60 households) in trying out the optimized THR recipes and SBCC messages in real-life settings. Structured weekly follow-up visits will be conducted with these households to document adherence, identify barriers, and capture user-driven modifications. Recipe acceptability, feeding quantity, preparation effort, and caregiver perceptions will be evaluated using observation checklists, meal logs, and structured feedback cards. Real-time feedback will be used to adapt recipes and refine SBCC messages around responsive feeding, dietary diversity, and hygiene.

The expected outputs from these activities include detailed documentation of recipe adherence and barriers across different nutritional status strata. Outputs will also encompass refinement of SBCC messages tailored to local beliefs, knowledge gaps, and motivational levers, as well as inputs for adjusting portion sizes and feeding frequency in relation to child nutritional status. All field investigators administering the questionnaires will undergo standardized training to ensure uniform application of tools and minimize interviewer bias.

#### 2.3.4 Synthesis of findings and stakeholder consultation

This 4-week period will focus on triangulating findings from the field and refining the integrated package through participatory stakeholder discussions. Findings from the TIPs cycles, including caregiver feedback, recipe adherence scores, and SBCC trial outcomes, will be consolidated and shared with local stakeholders and program functionaries. Stakeholder consultations will then be conducted with ICDS and health functionaries and community leaders to validate the feasibility and acceptability of the proposed recipes and messages, and to identify operational bottlenecks and solutions for informing the intervention. This process will help co-develop and finalize the integrated intervention package, ensuring alignment with ground realities and programmatic logistics. It will also help provide a stakeholder-endorsed implementation plan covering supply-side considerations (e.g., ingredient sourcing, shelf life, and logistics, etc.).

#### 2.3.5 Pilot testing of the integrated package

In the final step, the integrated package will be piloted for one complete monthly cycle to assess its operational viability and real-life consumption. The pilot will focus on monitoring supply chain functionality, actual consumption by target children, and implementation fidelity. THR packages will be distributed to the same ~60 households already engaged in Step 3.

A purposive sample of minimum 30 children aged 6–12 months and 30 children aged 13–36 months will be recruited from selected Anganwadi centres (AWCs), ensuring adequate representation from both rural and urban areas. Within the identified centres, efforts will be made to include all available cases of children with severe acute malnutrition (SAM) and moderate acute malnutrition (MAM). Efforts will also be made to ensure appropriate representation of Scheduled Caste/Scheduled Tribe (SC/ST) and non-SC/ST households during AWC and household selection.

Between 4–6 THR products will be evaluated during the pilot. Of these, 2–3 will be designed for normally nourished children and 2 for those with SAM or MAM. For each product, 2–3 recipe variations will be tested. Recipes will be served in age-appropriate portion sizes to younger children (6–12 months) and older children (13–36 months). Each product-recipe combination will be tested at least twice with adequate intervals, and the most consistent observations will be considered for analysis.

Over this one month, recipe preparation, feeding frequency, and child consumption will be monitored using daily food logs and periodic home visits. Acceptability, wastage, adherence, and practical challenges will be assessed, alongside feedback on implementation logistics from ICDS staff and caregivers.

Key indicators to be assessed include the quantity consumed per child per day across nutritional status strata (normal and MAM/SAM), barriers to preparation or uptake, and feedback on message retention and behaviour change initiation. The expected outputs from this phase include practical feasibility data for each recipe and SBCC component under program conditions, insights into the actual quantity of THR consumed by children across different malnutrition categories, and evidence to guide adjustments in ration size (currently set as double ration for malnourished children) to inform ICDS feeding guidelines. Ultimately, 2 to 3 THR products will be finalized to be used in the next study phase.

#### 2.3.6 Statistical analysis plan

Caregiver sensory evaluation scores obtained through Likert-type or hedonic scales will be treated as ordinal data. Summary statistics will be presented as medians and interquartile ranges. Differences in sensory preference scores across THR recipes will be assessed using non-parametric tests, including the Kruskal-Wallis test, as appropriate. Changes in acceptability and adherence scores across repeated TIPs cycles will be analyzed using the Friedman test.

Qualitative analysis will be performed by thematic coding of barriers and caregiver feedback. Content analysis of qualitative responses on cultural appropriateness and ingredient feasibility will be done.

### 2.4 Phase III: cluster randomized controlled trial to assess the effectiveness of the intervention package

#### 2.4.1 Study design and setting

A parallel two-arm, multi-centre cluster randomized controlled trial will be conducted in the six districts, each located in a different state, as outlined earlier, to evaluate the effectiveness of an intervention package including improved THR and SBCC strategies compared to the existing THR and SBCC strategy on the nutritional status of children aged 6–36 months. Areas within these districts where formative research or phase two activities have taken place will be excluded.

The effectiveness of the intervention package will be assessed at two levels:

(1) Individual (and household) level, focusing on children aged 6–36 months with special emphasis on the6-24 months age group, the critical window for growth and development.

(2) Population level, focusing on children aged 6–36 months.

A cluster randomized controlled trial (cRCT) will consist of two components to document the effectiveness at two levels, as mentioned above:

1. Individual level outcome—Longitudinal (cohort) approach: to study changes in the outcome indicators at an individual level in children aged 6–36 months. Eligible children aged 6–18 months will be recruited and followed for 18 months after initiation of the intervention, who turn 24–36 months old by the end of follow-up.
2. Population level outcome—Repeated cross-sectional approach: to study changes in the outcome indicators at the population level among children aged 6–36 months. All eligible children aged 6–36 months residing in the study area will be assessed at baseline, then at 6-month intervals (6, 12, and 18 months) and an endline at 24 months of initiation of the intervention implementation.

**Figure 4:**
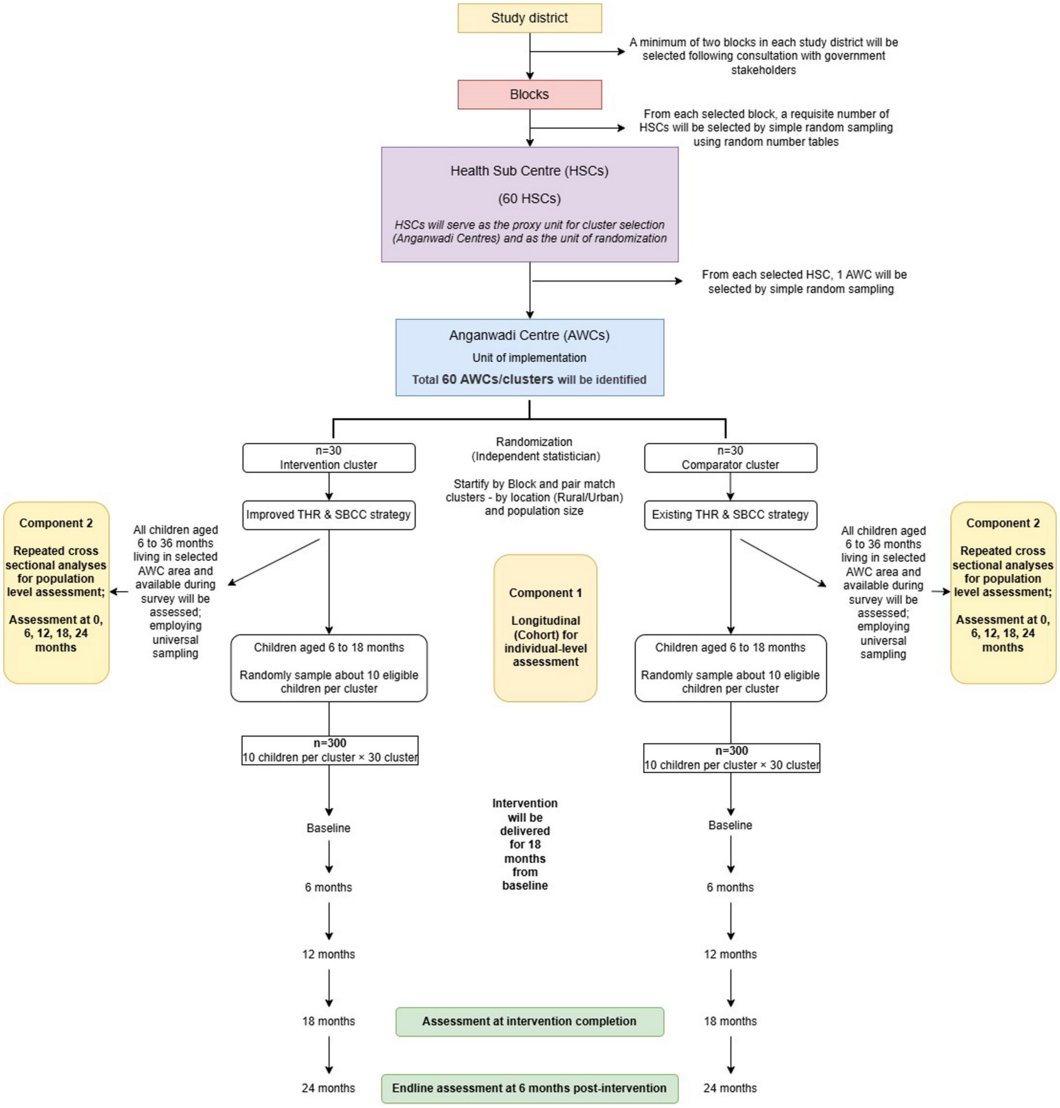
Schematic overview of the phase 3.

A schematic overview of phase 3 is illustrated in Figure 4.

#### 2.4.2 Study unit and clusters

In the study, anganwadi centres (AWCs) will serve as the unit of implementation. Considering the location of the AWCs in close proximity and the presence of multiple AWCs in the same village, which may pose a risk of contamination, Health Sub-Centres (HSCs)/Health and Wellness Centres (HWCs) will serve as the proxy unit for selecting the clusters (AWCs) and randomization. In each district, HSCs/HWCs will be randomly selected from the multiple blocks, with the number of HSCs/HWCs to be sampled from each block proportionate to the population of each block. A minimum of two blocks will be selected in each study district following discussions with government stakeholders. Requisite number of HSCs per block will be sampled using random number tables, ensuring representation across urban, rural, tribal, and diverse geographic constituencies. Under each selected HSC, one to two AWCs (depending on the population size, covering about 1,000 population to ensure availability of the desired number of children) will be randomly selected to form the study clusters.

To be eligible for inclusion, AWCs that are functional with both anganwadi worker (AWW) and anganwadi helper (AWH), actively available, will be considered. AWCs that are contiguous or located in hard-to-reach areas will be excluded.

#### 2.4.3 Study participants

All the children aged 6–36 months who live in the selected AWC area, registered with the AWC and regardless of their socioeconomic status, will be eligible to receive the THR and SBCC, according to the allocation arms. The quantity of THR that they will receive will be based on ICDS guidelines, considering both their age and nutritional status (normal, MAM, or SAM). There are no exclusion criteria. For the children not registered in the AWCs, the caregivers and household head will be counselled for registration in the AWC.

##### 2.4.3.1 Eligibility of the children for individual-level outcome assessment (cohort component)

Here, children under two years of age, belonging to the critical window of the first 1,000 days, will be targeted. Specifically, children aged between >6 and <18 months, of any gender and socioeconomic background, will be included. Eligibility will require that the child resides within the selected AWC area and is likely to remain there for the study duration, is registered with the AWC, and has a family willing to receive THR and SBCC services through the designated AWC. Children will be excluded if they have major congenital malformations or medical/surgical conditions (known/as reported by parents) that interfere with dietary intake, or if consent for participation is denied.

In each selected AWC, at least 10 children will be identified based on the eligibility criteria. Only one child per household shall be included in the outcome assessment. If more than one child in the eligible age group is available in the selected household, the younger child will be considered. It is anticipated that recruitment of the desired children across the clusters may take about 2–4 months. The primary outcome documentation for the cohort has been proposed at 18 months, expecting that the children aged 18 months will turn 36 months at the time of endline assessment.

##### 2.4.3.2 Eligibility of children for population-level outcome assessment (cross-sectional component)

Universal sampling will be employed for the cross-sectional component. All children aged 6–36 months living in the study area (AWC), of any gender and socioeconomic background, who are present during the survey, regardless of their AWC registration status and THR or SBCC usage, will be included in the repeated cross-sectional surveys. Children will be excluded if they have major congenital malformations or medical/surgical conditions (known/as reported by parents) that interfere with dietary intake, or if consent for participation is denied.

#### 2.4.4 Intervention

The intervention package will comprise an improved THR product and an SBCC strategy as prepared in phase 2. The improved THR will be prepared by an identified self-help group (a type of community-based organization), vendor, or manufacturer, based on the finalized composition of THR, cost considerations, and necessary regulatory approvals.

The intervention will be delivered primarily by anganwadi workers and anganwadi helpers from the ICDS system, supported by the Implementation Support team. Additionally, Accredited Social Health Activists (ASHAs), Auxiliary Nurse and Midwife (ANMs) from health functionaries will complement the efforts in intervention clusters as per need. Supervisory support will be provided by ICDS/Women and Child Development (WCD) and health officials at the block and district levels.

Participants in comparator clusters will continue to receive existing THR products and SBCC services as per the ongoing ICDS program without any change in composition and delivery.

Services in the intervention versus comparator arms are described in Supplementary Table 2.

#### 2.4.5 Study outcomes

The data for the longitudinal (individual-level outcome) and cross-sectional (population-level outcome) study components will be collected simultaneously at baseline (pre-intervention), and then at six-monthly intervals; i.e. at 6, 12, 18 and 24 months (endline). For the longitudinal or cohort component outcomes, the data collected at baseline, months 6, 12 and 18 months of intervention will be used to assess the key outcome and the trend in the cohort. Although the intervention will be implemented over 18 months, follow-up will continue until the endline at 24 months post-baseline. This extended follow-up period is aligned with the independent expert committee’s recommendations and allows for a more comprehensive assessment of the intervention’s effect, as key improvements in growth and nutritional outcomes are more likely to manifest over time.

For the cross-sectional component outcomes, the data collected between the baseline (month 0) and endline (month 24) will be used to assess the outcome and the trend (data at months 6, 12, and 18).

##### 2.4.5.1 Primary outcome

Change in weight-for-age z-score (WAZ), based on WHO growth standards, among children aged 6–36 months enrolled in the longitudinal component of the study, measured between baseline and 18 months of follow-up.

###### 2.4.5.2 Secondary outcomes

1. For children aged 6–36 months enrolled in the longitudinal component: nutritional status based on WAZ, HAZ, WHZ, and MUAC, IYCF practices, dietary and nutritional intake, water, sanitation and hygiene (WASH) practices, and health status.
2. For children aged 6–36 months assessed under the cross-sectional component: nutritional status based on WAZ, HAZ, and WHZ, and acceptance and usage of THR.
3. Other outcome indicators- Cost-effectiveness of the intervention: The incremental cost-effectiveness of implementing the intervention package will be compared with the existing THR and SBCC strategy in the study district.

Details of the outcome indicators to be assessed in Phase 3 are presented in Supplementary Table 3.

#### 2.4.6 Sample size

The sample size is calculated to detect a minimum mean difference of 0.3 in mean WAZ score between intervention and comparator arms, assuming a mean WAZ of −1.8 in the comparator group and a standard deviation of 1.0 in both groups (45, 46). With 80% power, 5% significance level, an intra-cluster correlation coefficient of 0.05, and an expected attrition rate of 20%, the estimated sample size is 300 per arm per site. This will be achieved through 30 clusters per arm, with an average of 10 children per cluster.

#### 2.4.7 Randomization and blinding

The unit of randomization will be HSC, as a proxy for the AWC. Sixty HSCs/HWCs from different blocks in the district will be identified using simple random sampling. Under each selected HSC, the eligible AWC, as per the eligibility criteria described in the earlier section, will be selected using simple random sampling for the implementation of study activities. To meet the required number of children in the eligible age groups in areas such as hilly/tribal/sparse habitation, more than one AWC (contiguous AWCs covering about 1,000–1,200 population) may be included, which will be considered as one cluster. For the larger villages/urban areas with more than one AWC, only one AWC (with a population of about 1,000–1,200) will be selected for inclusion in the study. While selecting the AWCs from the HSCs, it will be ensured that the AWCs selected from different HSCs are drawn from areas not involved in pilot studies, non-contiguous, and are located at a reasonable distance to minimize contamination.

Selected HSCs and 60 AWC units (clusters) will be randomized into the two arms by an independent statistician; 30 into the intervention arm and 30 into the comparison arm. Randomization will be stratified by the blocks included, such that every HSC/AWC randomized to the intervention arm will have an HSC/AWC randomized to the control arm from the same block. For randomization, pair matching of clusters will be done considering key parameters such as location stratum (urban–rural) and population size. Within each matched pair, one cluster will be randomized to the intervention arm and the other to the comparison arm. Within each selected AWC, 10 children will be randomly sampled from all eligible children in the cluster.

The allocation sequence will be generated by an independent statistician. The allocation code for each of the 60 clusters will be available only to the statistician and shared with the site investigator upon request, ensuring allocation concealment.

While blinding of investigators and participants to the intervention allocation is not possible, the data analysts will not be aware of the two study arms. There will be two separate study teams in the field that will be independently working: (1) an Implementation Support Team (IST) responsible for supporting the implementation of the planned packages in the intervention clusters, including training, supervision, product supply, monitoring, and periodic surveillance. The team will provide on-site support to anganwadi workers, document process indicators, and report any unintended negative outcomes. During field visits, team members will also interact with caregivers at the households and community members to understand their response and challenges; (2) Outcome Assessment Team (OAT) will be responsible for outcome assessment. The OAT will function independently of the IST, with restricted opportunities for interaction, and will be masked to intervention and comparison arms to minimize bias. The team will identify eligible households and conduct anthropometry measurements and data collection every six months (at baseline, 6, 12, 18, and 24 months) using appropriate tools for outcome assessments in both intervention and comparison clusters.

#### 2.4.8 Study procedures

The intervention will be implemented through a structured approach involving community engagement, capacity building of frontline workers, and systematic delivery of intervention components, supported by robust monitoring mechanisms.

##### 2.4.8.1 Community sensitization

Prior to commencement, orientation meetings will be held at each study site/cluster to orient key stakeholders, including panchayat members, Poshan Panchayat representatives, mothers’ groups, and the village health, sanitation and nutrition committee on study objectives, intervention package, activities, and the limited area approach, to secure community support and engagement.

##### 2.4.8.2 Training of anganwadi workers and helpers

In intervention clusters, AWWs and AWHs will undergo comprehensive training on the improved THR and delivery of the SBCC package. Training will include classroom sessions, on-field demonstrations, and on-the-job support using local language materials, case studies, group exercises, and videos. Supportive supervision from the study team will reinforce skills. Health functionaries in intervention clusters, including ASHAs and ANMs, will be oriented on improved THR and trained on SBCC strategies to complement ICDS activities. Joint sessions for ICDS and health staff may be organized as needed. Training will occur before intervention rollout, with planned refresher sessions at 3–4 and 6–8 months of initiation of the intervention, and additional sessions as needed to appropriately cater to the age-based changes in the dietary and behavioural practices.

AWWs and AWHs in the comparison clusters will not be given any additional training other than orientation about the study activities.

##### 2.4.8.3 Delivery of intervention package

The intervention package will be delivered through the existing ICDS platform. The home visit sessions of AWW will be utilized to deliver the integrated intervention package. AWWs in intervention clusters will distribute prescribed quantities of the improved THR fortnightly to eligible households (aligned with the existing THR supply guidelines for amount and frequency) and counsel caregivers and family members on its role in child growth. Improved THR packets will be supplied monthly to AWCs based on beneficiary numbers. Improved SBCC will have special emphasis during the home visits in addition to the improved THR distribution. The AWWs will be trained to be active listeners, more reflective and adaptive to the household needs, rather than just being directive and information sharing. AWWs will distribute Information, Education, and Communication (IEC) materials, including locally appropriate cookbooks in the local language, to help caregivers add variety to their preparations (taste/palatability), as well as promote IYCF and WASH practices. If any illness or sickness is identified during the child’s participation in the trial, the study team will ensure that the child is promptly referred to the nearest government healthcare facility for appropriate care.

##### 2.4.8.4 Health, early child development, and related activities

AWWs, ANMs, and ASHAs will continue routine health and early child development services in all clusters. In intervention clusters, caregivers will be counselled on immunization, WASH, care during childhood illnesses, and participation in Village Health and Nutrition Days (VHNDs), with iron and folic acid supplementation and deworming as per guidelines. In comparison clusters, the WCD and health functionaries will continue to provide the services as usual.

#### 2.4.9 Data collection

Data will be collected using appropriate tools to assess primary and secondary outcomes.

##### 2.4.9.1 Anthropometric assessment

Standard guidelines proposed by the WHO will be followed to assess anthropometric indicators (47). Weight will be measured using calibrated electronic weighing scales (seca 354) with a precision of 10gm. The length/height will be measured using a standardized measurement board/infantometer (seca 417)/stadiometer (seca 213) with a precision of 1 mm. The child’s MUAC will be measured using a non-stretchable tape (seca 201) with a precision of 1 mm. At one of the study sites, Hoshangabad, MUAC will be measured using standard colour-coded tapes supplied by UNICEF (48).

##### 2.4.9.2 Infant and young child feeding practices

Data on IYCF practices, including breastfeeding and complementary feeding, will be collected using a structured IYCF questionnaire, along with a 24-h dietary recall survey. WHO recommended core indicators of optimal breastfeeding, including exclusive breastfeeding till 6 months, continued breastfeeding for at least two years post-birth, along with complementary feeding practices, such as introduction of solid, semi-solid, or soft foods, minimum dietary diversity, minimum meal frequency, minimum acceptable diet, consumption of iron-rich or iron-fortified foods from postnatal 6 completed months and nutrient intake will be captured (49). Apart from a pre-tested questionnaire on IYCF practices, a two non-consecutive 24-h dietary recall survey will be administered on the mother/primary caregiver for assessing the dietary and nutrient intake of children enrolled for the longitudinal study. Respondents will include mothers and/or primary caregivers within the household. During this dietary survey, respondents will be asked to recall a detailed food intake of the child during the past 24 h. A standard food recall kit (measuring cups, spoons and food picture flip book) will be used by the interviewer for portion size estimation.

Household food security information will be collected using a questionnaire adapted from the food security and nutrition surveillance tool (50, 51).

##### 2.4.9.3 WASH-related knowledge, attitude, and practices (KAP)

Data on WASH will be collected using a KAP questionnaire developed based on a standard manual for WASH (3).

##### 2.4.9.4 Health status

The children’s morbidity data (e.g., diarrhoea and acute illnesses) will be collected using a 2-week recall questionnaire along with any hospitalization history. The vaccination data would be recorded based on the Essential Program on Immunization (EPI) schedule, verified from the immunization card or register with AWC/ASHA/ANM (52).

##### 2.4.9.5 Economic evaluation

The cost-effectiveness of the intervention will be conducted from a government provider perspective. The incremental economic cost of the intervention will be calculated using an ingredients approach, where each activity related to the intervention will be identified and costed. For example, for community engagement, there will be several stakeholder engagement meetings with panchayats members, community members, and mothers’ groups. Further, ANMs, ASHAs, AWWs and AWHs will be trained as part of the intervention. These activities from each study site will be documented in detail for cost analysis. Financial costs under each study activity will include actual expenses related to the intervention (e.g., expenses for staff training, SBCC materials, prescribed quantities of improved THR, etc.). Economic costs include financial costs and time costs related to various intervention activities. For example, hours spent by AWWs, AWHs during training, hours spent by AWWs during improved THR distribution and SBCC will be multiplied by the hourly wages of AWWs and AWHs to estimate the time costs of the activities. While actual expenses related to the intervention will be collected from financial records of each site, time spent data will be collected through interviews with key stakeholders such as AWWs and AWHs. Data will be collected alongside intervention development and roll-out using structured questionnaires. The total cost of the intervention site will be the sum of the financial and time cost of all activities related to intervention development and implementation. As the intervention will be compared with the existing THR and SBCC, the costs incurred for the intervention will be the incremental cost. Incremental effectiveness will be measured by examining the changes in weight-for-age z-score for children in the intervention and control arms. The cost-effectiveness ratio will be the incremental cost of the intervention/additional number of children with a mean difference of 0.3 in mean WAZ scores in the intervention arm as compared to the control arm.

##### 2.4.9.6 Sociodemographic data

Background characteristics of study participants, including the demographic and socioeconomic status, family members, reproductive and morbidity history of the mother, will be collected using a structured questionnaire (adapted from the National Family and Health Survey-5 household tool). The age of the child will be calculated based on the date of birth recorded from the birth certificate/record/discharge record. In the absence of any official birth records, the date of birth as reported by the mother/primary caretaker will be used. The socioeconomic status of the households will be captured using the Kuppuswamy scale.

In addition, a random sample of THR products will be analyzed for nutrient composition and bacterial content in NABL-accredited labs.

The participant timelines of data collection and analyses for different components are summarized in Table 3.

**Table 3:** Participant timelines (53) outlining enrollment, interventions, and scheduled assessments in Phase 3.

Activities and data components	Time points					
	Enrollment	Month 0 (baseline assessment)	Month 6	Month 12	Month 18	Month 24 (endline assessment)
Enrollment						
Eligibility screen	X					
Informed consent	X					
Randomization	X					
Intervention/comparator						
Improved THR and SBCC		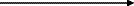				
Existing THR and SBCC		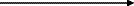				
Data collection						
I. Longitudinal component						
Socio-demographic information		X				X
Anthropometry indicators (weight, length/height, MUAC)		X	X	X	X	X
IYCF and nutritional practices, household food security		X	X	X	X	X
WASH practices		X	X	X	X	X
Health status		X	X	X	X	X
THR utilization		X	X	X	X	X
II. Cross-sectional component						
Anthropometry indicators (weight, length/height, MUAC)		X	X	X	X	X
THR utilization		X	X	X	X	X
Generic data						
Nutrient composition of improved THR		X				
Data collection for economic evaluation and analysis		X	X	X	X	X

Data collection team (OAT members) will receive rigorous training in anthropometric measurements according to standard protocols, as well as in the administration of study tools and data capture procedures. Additionally, intrarater and interrater reliability testing for anthropometry measurements will be undertaken across sites and data collectors. The data collected will be reviewed periodically to ensure accuracy and reduce missing outcome data.

#### 2.4.10 Data management

Data will be collected electronically using the REDCap application, an Electronic Data Capture (EDC) tool with built-in logic and consistency checks. To protect the confidentiality of participants, data collection and storage procedures will be regulatory compliant, restricting access to data on devices through passwords.

The data collectors will work in pairs in the field to administer questionnaires and measure children’s anthropometry. A field supervisor will be responsible for planning the data collection and coordinating data collection activities at each study site. As a routine quality control measure to maintain data integrity, repeat data collection will be conducted by field supervisors in a randomly selected 5% of the participants sampled across the clusters. The supervisor will report directly to the data management team. Data will be uploaded to the designated server daily by data collectors and checked weekly by the data management team. The errors or incomplete questionnaires identified will be sent back to the data collection teams to complete the questionnaire with participants. Data collectors will attempt to reach participants at least three times before participants are considered to be lost to follow-up for that data collection point. Monthly project management meetings will be held to track data quality throughout the trial. Only the data management team and investigators will have access to the complete dataset.

#### 2.4.11 Statistical analysis plan

Baseline characteristics of study participants, along with primary and secondary outcome variables, will be summarized by study groups using appropriate descriptive statistics depending on the measurement scale and data distribution. Continuous variables will be reported as means with standard deviations or medians with interquartile ranges, as appropriate, while categorical variables will be presented as frequencies and percentages, with 95% confidence intervals provided where applicable.

Dietary data captured through 24-h dietary recall will be utilized for calculating the ‘usual intake’, diet diversity, minimum meal frequency and minimum adequate diet. Using DietCal software (version 15.1.1), the reported amounts of raw foods in 24-h dietary recall will be entered, and converted to intake of different food groups, and macro- and micronutrients. The macronutrient and micronutrient intakes will then be transformed using a linear mixed-effect regression model. The values predicted using the linear mixed-effect regression model will be back-transformed to compute the “usual nutrient intake” (54). The estimated average requirement (EAR) cut-point method and full probability approach (for iron) will be used to estimate the prevalence of nutrient inadequacy of complementary feeding using the WHO recommendation outlined in Dewey et.al 2002 for children 6 months to 24 months and national recommendations for children above 24 months of age (55, 56). The raw dietary intake data will also be used to assess the dietary diversity, minimum meal frequency and minimum acceptable diet in these children (55).

The individual longitudinal assessment of WHO standardized anthropometric scores will be compared between the intervention and comparator groups using an intention-to-treat analysis (57). A linear mixed effects model with subject and cluster as random effects and an interaction term for time and intervention group will be performed. Separate analysis will be performed for WAZ, LAZ and WHZ. The stunting, wasting, and underweight prevalence among all THR beneficiaries in the recruited AWCs at the end of the intervention period will be compared between the intervention and comparator groups using log binomial regression, robust estimates of standard error accounting for clustering. Potential confounders (household food security, education, socioeconomic status, etc.) will be considered in the model. All episodes of illness or sickness occurring during the period of participation will be systematically recorded in the study data. For outcome assessment, illnesses with the potential to influence child growth trajectories will be accounted for during analysis through appropriate statistical adjustments and sensitivity analyses, as applicable.

All analyses will be performed within each site and across all sites by pooling the data. Analyses will be conducted using Stata statistical software (StataCorp LLC, College Station, Texas, United States), with two-sided tests and a significance threshold of *p* < 0.05.

#### 2.4.12 Data and safety monitoring

A Data and Safety Monitoring Board (DSMB) will be constituted for periodic review of safety-related issues. The DSMB will be an independent multidisciplinary group consisting of five voting members and two observers (from nutrition, paediatrics, public health/epidemiologist, clinical trials and biostatistics, in accordance with the national guidelines), ensuring no involvement in the study or any conflict of interest. During the study, any adverse events, whether solicited (e.g., acute gastritis, allergic reactions) or unsolicited, spontaneously reported or identified during study assessments, will be documented and reported to the DSMB for discussion and appropriate guidance.

## 3 Discussion

The present study is conceived with the underlying belief that delivering nutrient-rich, high-quality complementary foods alongside shaping caregivers’ behaviours can significantly promote healthy growth and development in children, contributing to the transformative vision of Sustainable Development Goal 2, which aims to improve nutrition and end all forms of malnutrition by 2030. This is particularly crucial during the 6-36-month period, a window when children are most vulnerable to wasting, underweight, and stunting, with long-term consequences for physical growth, cognitive development, and overall well-being (58–60). Evidence from low- and middle-income countries indicates that integrating high-quality complementary foods with culturally relevant SBCC can substantially improve IYCF practices and child growth outcomes (19, 61, 62). Yet, in India, there is limited empirical evidence on the effectiveness of locally tailored THR products combined with multi-level, context-specific SBCC interventions delivered at scale.

By integrating improved THR formulations with participatory SBCC strategies, this study aims to address key barriers such as low acceptability, inconsistent compliance, and lack of sustained behaviour change, which have likely to limit the impact of previous efforts. The approach emphasizes both on product optimization and effective communication strategies, recognizing that even the most nutritious THR will fail without community buy-in. Locally available, nutrient-dense foods will be incorporated into THR formulations to increase familiarity and acceptance, while SBCC efforts will focus on improving caregivers’ knowledge, attitudes, and feeding practices. Together, these elements are designed to bridge the gap between policy intentions and real-world adoption, ensuring alignment with regional dietary habits and socio-cultural norms.

The study’s strength lies in its comprehensive methodology. The inclusion of six states representing diverse geographic, cultural, and dietary contexts will enhance the generalizability of the findings across varied Indian settings. The phased, mixed-methods approach enables iterative refinement of both THR formulations and SBCC strategies, ensuring cultural alignment and implementation feasibility. The cluster randomized controlled trial will provide robust evidence on effectiveness, and the inclusion of both longitudinal and repeated cross-sectional components will enable the capture of individual growth trajectories as well as community-wide trends. The learnings from implementation will be essential for informing scale-up.

The findings from this study will generate actionable evidence on what works, what does not, and why. These insights will guide the development of more effective, sustainable strategies to improve complementary feeding practices and child nutrition in India.

## 4 Ethics and dissemination

The protocol has been reviewed by a Technical Advisory Committee, and ethical clearance has been obtained from the Institutional Ethics Committees of all implementing institutions across the study sites. Prior to commencement, the study has been prospectively registered with the Clinical Trials Registry of India. Any protocol amendments will be submitted to the respective ethics committees and updated in the clinical trial registry. Written informed consent will be obtained from the parents of eligible children prior to recruitment and data collection. Additionally, at the community level, group consent will be sought at the village level before initiating study activities in the selected clusters. Appropriate administrative approval from state and district authorities will be secured for study implementation. All participant data will be anonymized and stored securely, with access restricted to the research team to ensure confidentiality. Study findings will be disseminated to participants, local communities, program managers at the district, state, and national levels, and policymakers, in addition to being shared through peer-reviewed publications and scientific conferences or meetings.

Details of the ethics committee at each site are as below:

1. Institutional Human Ethics Committee, AIIMS Bhopal, Madhya Pradesh, India; reference no: IHEC-LOP/2024/P24/044.
2. Institutional Ethics Committee, AIIMS Bhubaneshwar, Odisha, India; reference no: T/EMF/Biochem/2023-24/182.
3. Institutional Ethics Committee, Datta Meghe Institute of Higher Education and Research, Wardha, Maharashtra; reference number: DMIHER (DU)/IEC/2024/105.
4. Institutional Ethics Committee, ICMR-National Institute of Nutrition, Hyderabad, Telangana, India; Protocol No: 07/II/2024, valid upto 21st August, 2025; protocol no: NIN/IEC/2025/8/CR/002, valid upto 18th August, 2026.
5. Institutional Ethics Committee, St. John’s Medical College, Bangalore, Karnataka, India; reference no: 155/2024.
6. The George Institute Ethics Committee (TGIEC), India; project no 11/2024.

## Abbreviations

ANM: Auxiliary Nurse and Midwife
ASHA: Accredited Social Health Activist
AWC: Anganwadi Centre
AWH: Anganwadi Helper
AWW: Anganwadi Worker
cRCT: Cluster Randomized Control Trial
CHWs: Community Health Worker
DSMB: Data Safety Monitoring Board
EDC: Electronic Data Capture
EPI: Essential Program on Immunization
FGD: Focus Group Discussion
FSSAI: Food Safety and Standards Authority of India
HAZ: Height-for-age z score
HBYC: Home-Based Care for Young Child
HSC: Health Sub-Centre
HTTPS: Hypertext Transfer Protocol Secure
HWC: Health and Wellness Centre
ICDS: Integrated Child Development Services
ICMR: Indian Council of Medical Research
IDI: In-Depth Interview
IEC: Information, Education, and Communication
IQR: Interquartile Range
IST: Implementation Support Team
IYCF: Infant and Young Child Feeding
KABP: Knowledge, Attitudes, Beliefs, and Practices
KAP: Knowledge, Attitudes, and Practices
KII: Key Informant Interview
LAZ: Length-for-age z score
MAM: Moderate Acute Malnutrition
MUAC: Mid-Upper Arm Circumference
MUW: Moderate Underweight
NABL: National Accreditation Board for Testing and Calibration Laboratories
NECCTAR: Nutritional Status Enhancement of Children through Behaviour Change Communication and Take-Home Ration in India
NITI: National Institution for Transforming India
OAT: Outcome Assessment Team
PHC: Primary Health Centre
THR: Take Home Ration
TIPs: Trials of Improved Practices
SAM: Severe Acute Malnutrition
SBCC: Social Behaviour Change Communication
SC: Schedule Caste
SD: Standard deviation
SDGs: Sustainable Development Goals
SHG: Self Help Group
SNP: Supplementary Nutrition Programme
ST: Schedule Tribe
SUW: Severe Underweight
VHND: Village Health and Nutrition Day
WASH: Water, Sanitation and Hygiene
WAZ: weight-for-age z score
WCD: Women and Child Development
WHO: World Health Organization
WHZ: Weight-for-height z score
WLZ: Weight-for-length z score
