# Supplementary material for: A cluster randomized trial protocol to evaluate the effectiveness of an integrated package of improved take-home foods complemented with social and behaviour change communication strategies to improve nutritional status in children aged 6–36 months in six states of India: NECCTAR trial
Source: Front Nutr. 2026 Mar 12;13:1712806. doi: 10.3389/fnut.2026.1712806 (PMC13019731; doi:10.3389/fnut.2026.1712806)
Supplement: Supplementary file 1 [file Table_1.docx]

**Supplementary Tables**

**Table 1: Procedure to optimize THR product in phase 2**

| **Activities** | **Details** |
| --- | --- |
| Selection of Ingredients | • Commonly available and consumed foods as identified in the formative phase  • Identifying ingredients from existing THRs suitable for reformulation • Final selection will be based on nutritive value, local availability, and cost. |
| THR Formulation and Production | • 2–3 most acceptable, nutritious food blends will be formulated (teams may use optimization software to develop the blends).  • Additional costs for production, packaging, and transportation will be considered. • Formulations will meet energy density and nutrient requirements as per the 2023 Government of India Gazette and WHO dietary diversity guidelines. • Sensory evaluation will be conducted by 15–20 semi-trained institutional panelists per site. • Revisions will be made based on expert consensus if required. • Suitable food manufacturing units (SHGs/vendors/enterprises/industry) will be identified with necessary approvals, ensuring standardized QA/QC procedures. |
| Determination of Quality and Stability of Improved THRs | • Chemical and nutritional properties (pH, water activity, moisture, protein, fat, energy, carbohydrates, selected vitamins, and minerals) will be determined using standard procedures. • Microbiological testing will include total viable count, coliforms, Escherichia coli, Staphylococci, etc. • Accelerated shelf-life testing will be conducted in accredited laboratories. • All analyses will be done in FSSAI-certified laboratories; 10% of the samples will be sent to a second FSSAI-certified laboratory. |
| Packaging and Labelling | • Design, package size, label information, and messaging will comply with FSSAI standards and guidelines. • Inputs from the formative phase will be incorporated into packaging and labelling.  •Packaging to be distinct from existing THR packets for easy identification and distribution  •Improved THRs may be produced locally at each site or centrally, based on the final THR formulation per site. |
| Standardization of age-appropriate, child-friendly Recipes | • Age-appropriate recipes will be standardized using improved THR blends. • Recipe booklets and videos will be developed for recipes using home foods |

**Table 2: Services in the Intervention versus Comparator arms**

| **Component** | **Intervention Arm** | **Comparator Arm** |
| --- | --- | --- |
| THR distribution | Improved THR (Take-home ration) packets/products with locally appropriate cookbooks/recipes in the local language(s) to help caregivers with added variety(ies) to the preparations | Existing THR supply continues as per the current ICDS program. |
| SBCC strategy | Improved SBCC to have special emphasis during the home visits, with AWWs trained in enhanced SBCC delivery.  IEC materials will be distributed, focusing on complementary feeding, IYCF, and WASH. | Existing SBCC strategies at AWCs continue as usual. |
| Health, early child development and other activities | Counselling to be provided on immunization, appropriate WASH practices, care-seeking during childhood illness, and participation in VHNDs.  Nutritional supplementation (iron, folic acid) and deworming to be provided as per existing guidelines, with counselling of WCD and health functionaries for adoption. | Routine counselling and supplementation to be provided by the AWC team and other stakeholders as per the existing government program. |
| Support to anganwadi workers and helpers | Training and support to be provided to AWWs and functionaries for effective implementation. | Routine refresher training and support by the WCD department system. No additional training or support is to be provided. |

**Table 3: Primary and secondary outcomes to be assessed under the longitudinal and cross-sectional components**

| **No** | **Outcomes** | **Indicators** |
| --- | --- | --- |
| **1** | **Primary Outcome** | - Change in weight-for-age z-score (WAZ) between baseline and 18 months of follow-up, based on WHO growth standards, among children aged 6–36 months enrolled in the longitudinal component of the study. |
| **2** | **Secondary Outcome** |  |
| **2.1** | **For the children aged 6-36 months in the longitudinal component** |  |
|  | Anthropometry indicators  (Weight, length/height, MUAC) | - Height/length-for-age z score, HAZ - Weight-for-height z score, WHZ - % of children with wasting (WHZ<-2 SD) - % of children with stunting (HAZ <-2 SD) - % of children with underweight (WAZ <-2 SD) - % of children with moderate underweight (MUW) (-3 SD ≤ WAZ < -2 SD) - % of children with severe underweight (SUW) (WAZ <-3 SD) - % of children with moderate acute malnutrition (MAM) (-3 SD ≤ WHZ < -2 SD) - % of children with severe acute malnutrition (SAM) (WHZ<-3 SD) |
|  | IYCF practices, minimum dietary diversity, and nutrient intake | - % of children who received an appropriate quantity and frequency of complementary feeding - % of children with continued breastfeeding till two years of age - % of children compliantwith the THR feed for the age - % of caregivers with appropriate knowledge, attitude, practice and behaviour related to the IYCF - % of children with minimum dietary diversity (for children till 23 months of age) - % of children meeting the Estimated Average Requirement for macro- and micronutrients (assessed using 24-hour dietary recall) |
|  | WASH practices | - % of households with access to clean drinking water - % of households with access to safe sanitation and defecation - % of households with good child excreta handling practices - % of households with good general hygiene, handwashing practices |
|  | Health status | - % of children with acute illnesses (fever, cold/cough, diarrhoea) with appropriate care-seeking practices (last 2 weeks) - % of children with full immunization status for the age |
|  | THR acceptance and usage | - % of children given the THR feed appropriate for their age |
| **2.2** | **For the children aged 6-36 months in the cross-sectional component** |  |
|  | Anthropometry indicators (Weight, length/height, MUAC) | - Weight-for-age z score, WAZ - Height/length-for-age z score, HAZ - Weight-for-height z score, WHZ - % of children with wasting (WHZ<-2 SD) - % of children with stunting (HAZ <-2 SD) - % of children with underweight (WAZ <-2 SD) - % of children with moderate underweight (MUW) (-3 SD ≤ WAZ < -2 SD) - % of children with severe underweight (SUW) (WAZ <-3 SD) - % of children with moderate acute malnutrition (MAM) (-3 SD ≤ WHZ < -2 SD) - % of children with severe acute malnutrition (SAM) (WHZ<-3 SD) |
|  | THR acceptance and usage | - % of children given the THR feed appropriate for their age |
| **3** | **Other outcome indicators** | - Cost-effectiveness of the intervention |

Body composition: Body composition measurement, although it would have been ideal, has not been proposed in the study due to the budget constraints.
